# Safety, Pharmacokinetics, and Pharmacodynamics of a Single Bolus of the γ-aminobutyric Acid (GABA) Receptor Potentiator HSK3486 in Healthy Chinese Elderly and Non-elderly

**DOI:** 10.3389/fphar.2021.735700

**Published:** 2021-08-27

**Authors:** Xiaojiao Li, Deming Yang, Qianqian Li, Hong Wang, Meng Wang, Pangke Yan, Nan Wu, Fangqiong Li, Shiping Ma, Yanhua Ding, Jingrui Liu, Hushan Wang

**Affiliations:** ^1^ Phase I Clinical Trial Unit, First Hospital, Jilin University, Jilin, China; ^2^ Jilin Medical Products Administration, Jilin, China; ^3^ Haisco Pharmaceutical Group, Chengdu, China; ^4^ Department of Anesthesiology, First Hospital, Jilin University, Jilin, China

**Keywords:** efficacy, elderly volunteers, pharmacokinetics, safety, HSK3486

## Abstract

**Background:** The present clinical trial investigated the potential influences of dosage and age on the pharmacokinetic properties and safety profile of HSK3486, and whether any adjustment in dosing regimen is necessary in elderly patients.

**Methods:** Twenty-four elderly participants (65–73 years) were apportioned to three equal cohorts to receive a single IV bolus of 0.2, 0.3, and 0.4 mg/kg HSK3486, respectively. An additional control group comprised eight non-elderly participants (21–44 years), who each received a single IV bolus dose of 0.4 mg/kg. Safety was assessed throughout the study, and the clinical effects were assessed based on modified observer’s assessment of alertness/sedation and bispectral index (BIS) monitor. Pharmacokinetic parameters were calculated.

**Results:** The rates of drug-related adverse reactions among the elderly groups were a little higher than that of the non-elderly, and were slightly higher in the elderly receiving 0.4 mg/kg compared with the elderly given lower doses. The pharmacokinetic characteristics of 0.4 mg/kg HSK3486 in the elderly and non-elderly were comparable. The time to recovery was similar in elderly 0.3 mg/kg, elderly 0.4 mg/kg and non-elderly 0.4 mg/kg groups. In the elderly 0.2 mg/kg group, the time to loss of consciousness was a little longer, and the time to recovery was shorter, relative to the other three groups.

**Conclusions:** Administration of 0.3 mg/kg to the elderly and 0.4 mg/kg to the non-elderly were similarly efficacious. A dose of HSK3486 of 0.3 mg/kg may be chosen for clinical use in elderly patients.

## Introduction

Propofol is one of the most widely used intravenous anesthetics in clinical practice. It is always used in the induction and maintenance of general anesthesia for surgery, as well as auxiliary outpatient surgery, due to its quick onset, short duration of anesthesia induction, and quick recovery of patients (Adler, 2017; Sahinovic et al., 2018). However, propofol also has obvious disadvantages, including injection pain, decreased diastolic blood pressure and mean arterial blood pressure, respiratory depression, and contraindications in patients with lipid metabolism disorders (Picard and Tramèr, 2000; Adam et al., 2004; Marik, 2004; Adler, 2017; Sahinovic et al., 2018).

**FIGURE 1:**
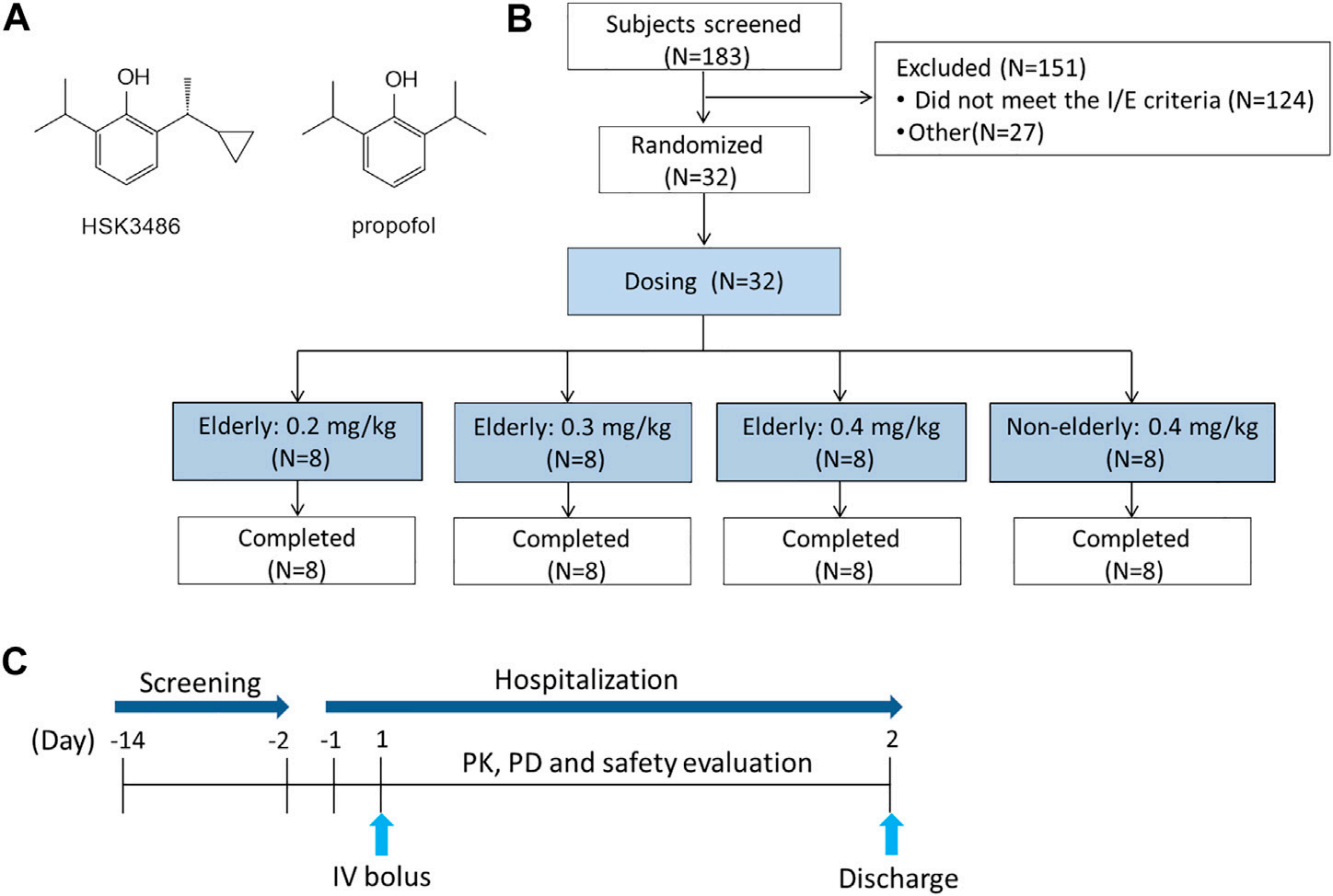
Relevant schematics. **(A)** Chemical structures of HSK 3486 and propofol. **(B)** Study design. **(C)** Flow chart of the study.

HSK3486, a gamma-aminobutyric acid (GABA) receptor potentiator similar to propofol, is a new candidate drug for the intravenous induction and maintenance of anesthesia in clinical practice (Figure 1). Compared with propofol, HSK3486 causes significantly less injection pain and hypotension (Qin et al., 2017; Wei et al., 2017; Bian et al., 2020). Several clinical trials (phase I to III) concerning HSK3486 have been completed in China and Australia, with overall more than 500 subjects enrolled, including those healthy or undergoing colonoscopy, gastroscopy, or elective surgery. Results indicate that the effective characteristics of HSK3486 are consistent with that of propofol, but the former can achieve the same anesthesia depth with 20–25% the dosage, and is associated with less inhibition of cardiac function and more stable hemodynamics. The plasma concentration of HSK3486 undergoes 3-phase elimination, with half-lives of initial, second, and terminal elimination phases of 2.0, 34.9, and 6.2 h, respectively, that are comparable with propofol (Bian et al., 2020; Ludbrook et al., 2021) (NCT04054063, NCT04037657, NCT04033939, NCT03773835, NCT03698617, NCT03709056, NCT03773042, NCT03808844, NCT03674008).

As the aged population increases, more and more patients scheduled for general surgery are elderly. Physiological changes accompany aging that make elderly patients more sensitive to anesthetics, in terms of induction dose and acute toxicity. These changes affect cardiac structure and function; liver, kidney, and gastrointestinal systems; neuroendocrine responses; and body composition (Macdonald et al., 1993; Beaufrère and Morio, 2000; Grandison and Boudinot, 2000; Vuyk et al., 2001; Orr and Chen, 2002; McLean and Le Couteur, 2004; Schmucker, 2005; Wynne, 2005; Corsonello et al., 2010; Perico et al., 2011). Propofol dosages and rates of administration in the elderly are generally reduced to diminish unwanted side effects, based on empirical findings (Vuyk et al., 2001). Standardized dosing schemes for HSK3486 in the elderly population are not yet available.

The present clinical trial investigated the potential influences of dosage and age on the pharmacokinetic properties and safety profile of HSK3486, and whether any adjustment in dosing regimen is necessary in elderly patients.

## Methods

### Ethics

This clinical study was conducted at Jilin University First Affiliated Hospital-Phase I Clinical Research Center, Changchun City, China. Ethical approval for this study was provided by the Ethics Committee at Jilin University First Affiliated Hospital-Clinical Research Institute, Changchun, China (Chairperson Prof Junqi Niu) on November 11, 2019 (19Y214-001). This clinical trial (registration No.: NCT04197661, https://clinicaltrials.gov/) was conducted in accordance with the World Medical Congress Declaration of Helsinki and Good Clinical Practice guidelines. All study subjects provided written informed consent.

### Subjects

Eligible subjects were ASA (American Society of Anesthesiologists) physical status I or II, without risk of a difficult airway (modified Mallampati score I or II) and Allen test-negative. The study population comprised healthy volunteers, non-elderly (18–65 years) and elderly (≥65 years), men and women with a body mass index (BMI) between 18 and 30 kg/m^2^, with no clinically significant abnormal findings on physical examinations, medical history, or clinical laboratory test results during screening. Subjects were excluded if they had a history of long-time smoking; alcohol or drug abuse; positive findings for hepatitis B or C virus, syphilis, or human immunodeficiency virus; or participated in an investigational drug study within the previous 3 months. Female subjects were not pregnant or lactating, and were using medically acceptable forms of birth control or were non-fertile.

### Study Design

This was a single center, randomized, open-label, parallel-group study of HSK3486. The 24 elderly participants were apportioned into three equal cohorts, who respectively received a single IV bolus dose of 0.2, 0.3, or 0.4 mg/kg HSK3486. A control cohort consisted of eight non-elderly participants who received a single IV bolus dose of 0.4 mg/kg. Since the tolerability of HSK3486 in the elderly is unknown, the 0.2 mg/kg HSK3486 in elderly group was conducted first, and dose escalation was dependent on the safety and tolerability of the previous doses, the last group was the 0.4 mg/kg HSK3486 in non-elderly. In each group, the eligible subjects were assigned the randomization numbers according to the order of screening number. The doses were chosen based on previous phase I-III studies of HSK3486 (Figure 1).

Subjects entered the study site and fasted for at least 8 h overnight before the study drug was administered. Water was not permitted from 2 h before dosing. Before dosing, all subjects were transferred to a dedicated treatment room with monitoring equipment, emergency equipment, and drugs to treat potential adverse events. A certified anesthesiologist was present during the drug administration until the subject recovered consciousness and had normal cardiovascular and respiratory function. Monitoring of vital signs was started 30 min before dosing, including 3-lead electrocardiogram (ECG), non-invasive blood pressure (BP) measurements, and pulse oximetry. Additionally, a bispectral index (BIS) monitor (BIS VISTA™ monitor; Aspect Medical Systems, Norwood, MA, United States) was used. Beginning 2 min before dosing until the end of drug administration, additional oxygen was delivered via nasal prongs. The oxygen flow was adjusted to 1–3 L/min until the subjects were fully awake. When fully awake after dosing, subjects were able to eat and drink. Subjects were discharged on day 2 after safety evaluation.

### Safety

Safety and tolerability were evaluated according to the National Cancer Institute Common Terminology Criteria for the Classification of Adverse Events, 5.0. Safety was assessed by monitoring AEs. Vital signs included systolic and diastolic blood pressure (SBP, DBP, respectively), mean arterial BP, heart rate, body temperature and respiration rate. Other monitoring included continuous 3-lead cardiac monitoring, 12-lead ECGs, continuous pulse oximetry, physical examination, and clinical laboratory tests (biochemistry, hematology, urinalysis, and coagulation). The injection pain was also evaluated. AEs and severity were reported at the time of incidence, and the association of AEs to the study drug was recorded. Sedation-related adverse events (hypotension, bradycardia, apnea, and hypoxia) were given special attention.

### Pharmacokinetic Analysis

Arterial pharmacokinetic samples (3 ml each) were collected at 0 h (pre-dose), and at 1, 2, 4, 8, 15, and 30 min, and 1 h after the start of the infusion. Venous blood samples were collected at 2, 3, 4, 6, 8, 12, and 24 h post-dose. In addition, 5 ml arterial blood was collected 1 min after administration and used for the determination of protein binding rate.

Blood samples were collected into K_2_EDTA-containing tubes and centrifuged at 1700 × *g* and 4°C for 10 min. The plasma was separated and stored in polypropylene tubes in two equal aliquots at −80°C until analysis.

Plasma concentrations of HSK3486 were determined via a validated liquid chromatography with tandem mass spectrometry (LC-MS-MS) method with a Shimadzu LC-30AD (Shimadzu, Kyoto, Japan) equipped with a Sciex Triple Quad 6,500 + quadrupole MS detector (AB Sciex, Toronto, Ontario, Canada). The calibration range of the assays for HSK3486 was 5.0–5,000 ng/ml. The calibration range was 0.8–2000 ng/ml for HSK3486 plasma protein binding rate determination. Accuracies for HSK3486 determination and plasma protein binding rate were, respectively, −2.8–2.7% and 0.0–8.3%, and the precision of each was within a coefficient of variation (CV) of 3.0%.

### Pharmacodynamic Analysis

The clinical hypnotic-anesthetic drug effect of HSK3486 was evaluated according to BIS monitoring, and assessments of Modified Observer’s Assessment of Alertness and Sedation (MOAA/S) scores. BIS was measured continuously using an integrated BIS Vista monitor. The BIS scores were recorded pre-dose and every minute for 60 min. The MOAA/S scores were recorded pre-dose and every minute within 5 min post-dose, and every 2 min thereafter until full recovery. Subjects were considered recovered when three consecutive MOAA/S scores of five were obtained.

### Statistics

According to the National Medical Products Administration (NMPA), for pharmacokinetics studies a sample size of 8–12 subjects is sufficient for an evaluation of pharmacokinetic characteristics. Thus, in the current study eight subjects were assigned in each cohort. All statistical tests were performed using SAS 9.4 software. Continuous variables were described as the mean (standard deviation) or median (minimum, maximum). Counting and grading data are shown as n (%). Pharmacokinetic parameters were calculated using non-compartmental analysis techniques with WinNonLin, version 7.0 (Certara, Princeton, NJ, United States). Results are presented as mean (SD). Time-to-peak (T_max_) is shown as median (range). One-way analysis of variance (ANOVA) and Least Significance Difference (LSD) was used for comparison between groups.

A power model was used to explore the dose proportionality of a single dose of HSK3486 among elderly subjects. The pharmacokinetic-pharmacodynamic association was investigated by linear regression.

## Results

### Subjects

Overall, 183 subjects were screened, and 32 (8 for each group) were enrolled and all completed the study (Table 1). The age range for elderly and non-elderly participants was 65–73 years and 21–44 years, respectively. According to the eligibility criteria, the ASA physical status was I or II, modified Mallampati score was I or II, and the Allen test was negative. The three elderly cohorts were statistically comparable in height, weight, BMI, creatinine clearance (CrCl), and age.

**TABLE 1:** Subjects’ demographic and baseline characteristics.

	Elderly 0.2 mg/kg	Elderly 0.3 mg/kg	Elderly 0.4 mg/kg	Non-elderly0.4 mg/kg	Total
	(*N* = 8)	(*N* = 8)	(*N* = 8)	(*N* = 8)	(*N* = 32)
Age (yr)	—	—	—	—	—
Mean (SD)	67.1 (2.70)	67.6 (1.77)	67.4 (2.67)	35.5 (7.65)	59.4 (14.62)
Median (Min, Max)	66.5 (65, 73)	68 (65, 70)	66.5 (65, 73)	38.5 (21, 44)	66 (21, 73)
Sex, n (%)	—	—	—	—	—
Male	7 (87.5)	7 (87.5)	4 (50.0)	5 (62.5)	23 (71.9)
Female	1 (12.5)	1 (12.5)	4 (50.0)	3 (37.5)	9 (28.1)
Height, cm, mean (SD)	164.2 (3.65)	164.5 (5.00)	157.3 (5.70)	165.3 (9.97)	162.8 (7.02)
Weight, kg, mean (SD)	66.6 (7.50)	65.8 (7.63)	59.0 (8.36)	62.7 (5.85)	63.5 (7.65)
BMI, kg/m^2^, mean (SD)	24.8 (3.01)	24.4 (2.39)	23.6 (2.07)	23 (1.85)	23.9 (2.35)
CLcr, mL/min, mean (SD)	84.2 (15.9)	81.9 (8.23)	71.7 (11.2)	114.2 (15.4)	88.0 (20.4)
Airway evaluation	—	—	—	—	—
I	5 (62.5)	5 (62.5)	2 (25.0)	6 (75.0)	18 (56.3)
II	3 (37.5)	3 (37.5)	6 (75.0)	2 (25.0)	14 (43.8)
Allen’s test	—	—	—	—	—
Negative	8 (100)	8 (100)	8 (100)	8 (100)	32 (100)
Positive	0	0	0	0	0
ASA physical status	—	—	—	—	—
I	4 (50.0)	7 (87.5)	3 (37.5)	7 (87.5)	21 (65.6)
II	4 (50.0)	1 (12.5)	5 (62.5)	1 (12.5)	11 (34.4)

BMI, body mass index; ClCr, creatinine clearance rate.

ClCr is based on the Cockcroft–Gault calculation at screening: male ClCr = [140−age (y)] × weight (kg)/[0.818 × SCr (μmol/L)]; female ClCr = 0.85 × male ClCr.

### Safety and Tolerability

There were 13 adverse events experienced among 10 elderly participants (Table 2). Ten adverse events among seven subjects were possibly related to the drug administration. There was no drug-related adverse event in the non-elderly 0.4 mg/kg group (Table 2). The drug-related adverse events among the elderly consisted of the following: hyperglycemia and hematuria (0.2 mg/kg); hypotension and respiratory depression (0.3 mg/kg); decreased BP; respiratory depression; supraventricular arrhythmia; first-degree atrioventricular block; hyperglycemia; and hypoalbuminemia (0.4 mg/kg). The drug-related adverse events were low in incidence, each occurring in one to two subjects only. The rates of drug-related adverse reactions among the elderly groups were a little higher than that of the non-elderly, and were slightly higher in the elderly receiving 0.4 mg/kg compared with the elderly given lower doses. All the adverse events were mild-to-moderate in severity, and fully resolved within a few hours to a few days after onset (except for one subject lost to follow-up). No adverse event required medical treatment, except for one subject with decreased blood pressure in the elderly 0.4 mg/kg group, which was treated with noradrenaline. There were no serious adverse event and no death occurred during the study. No adverse event led to withdrawal from the study. No subject reported notable pain on injection.

**TABLE 2:** Summary of adverse events.

	Elderly 0.2 mg/kg (*N* = 8)	Elderly 0.3 mg/kg (*N* = 8)	Elderly 0.4 mg/kg (*N* = 8)	Non-elderly 0.4 mg/kg (*N* = 8)	Total (*N* = 32)
—	n (%)	n (%)	n (%)	n (%)	n (%)
Total	3 (37.5)	2 (25.0)	4 (50.0)	1 (12.5)	10 (31.3)
Hyperglycemia	2 (25.0)	0 (0)	1 (12.5)	0 (0)	3 (9.4)
Hypoalbuminemia	0 (0)	0 (0)	2 (25.0)	0 (0)	2 (6.3)
Respiratory depression	0 (0)	1 (12.5)	1 (12.5)	0 (0)	2 (6.3)
Supraventricular arrhythmia	0 (0)	0 (0)	1 (12.5)	0 (0)	1 (3.1)
First degree atrioventricular block	0 (0)	0 (0)	1 (12.5)	0 (0)	1 (3.1)
Blood pressure decreased	0 (0)	1 (12.5)	1 (12.5)	0 (0)	2 (6.3)
Hematuria	1 (12.5)	0 (0)	0 (0)	0 (0)	1 (3.1)
Anemia	0 (0)	0 (0)	0 (0)	1 (12.5)	1 (3.1)

n: the subject number; %: the percentage of the AE.

**FIGURE 2:**
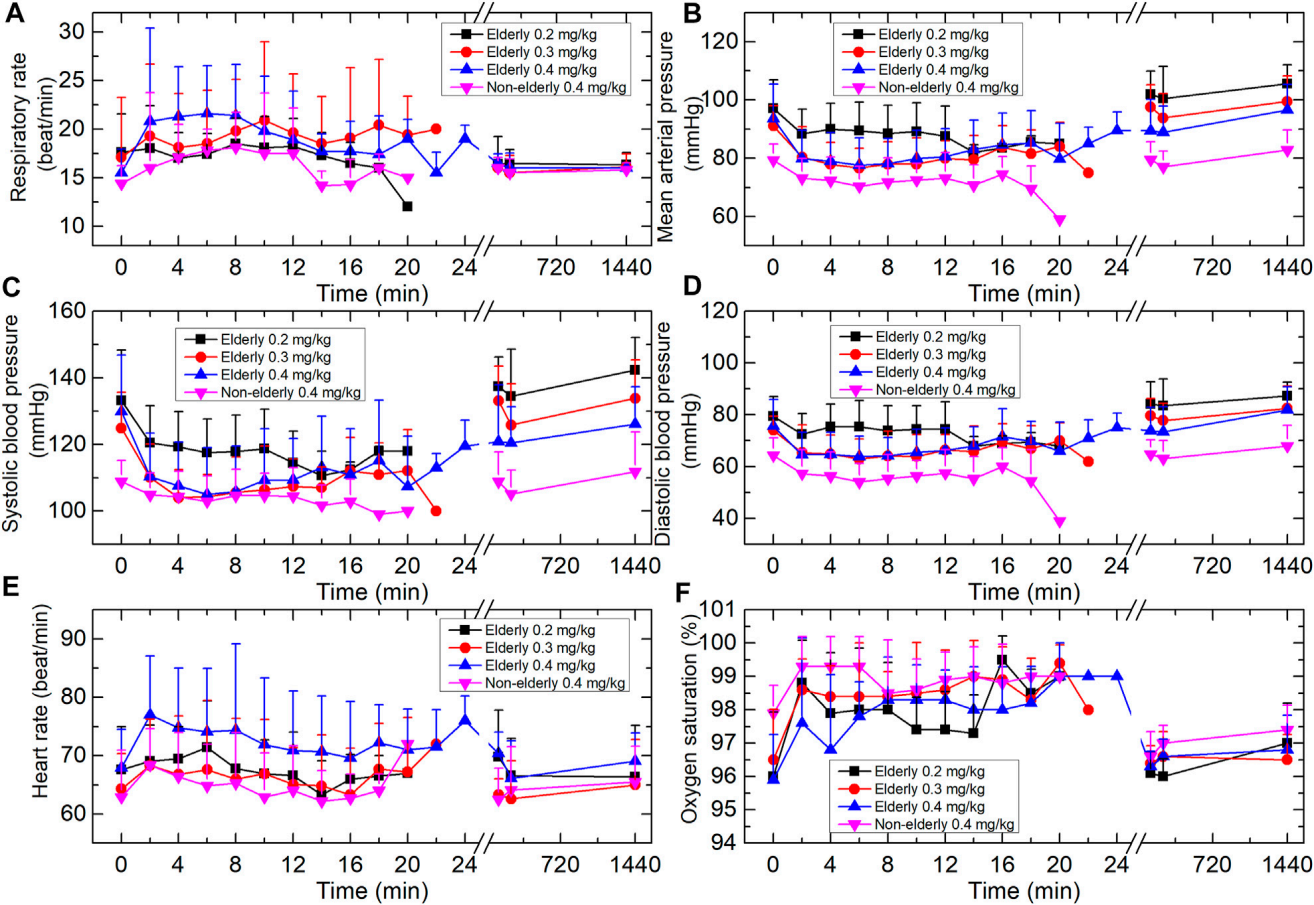
Vital signs of the four groups. **(A)** Respiration rate. **(B)** Mean arterial pressure. **(C)** Systolic blood pressure. **(D)** Diastolic blood pressure. **(E)** Heart rate. **(F)** Oxygen saturation (SpO2). Data are reported as mean + standard deviation.

Respiration rate tended to increase over time in a dose-dependent manner, with a maximum increase of ∼40% from baseline in the elderly 0.4 mg/kg group (Figure 2). There was no change in respiration rate that was considered clinically significant. Two subjects in the elderly 0.3 and 0.4 mg/kg groups experienced a transient respiratory depression; however, arterial oxygen saturation remained relatively unaffected.

During the study, BP tended to decrease over time. One subject in the elderly 0.3 mg/kg group experienced transient hypotension (SBP was 87 mmHg and lasted 4 min). One subject in the elderly 0.4 mg/kg group experienced a transient hypotension (DBP <60 mmHg). None of these subjects had clinical signs of hypoperfusion. No other subjects displayed a clinically significant change in BP. The mean heart rate of each cohort increased by a maximum of 6–14%. These changes were not considered clinically significant.

### Pharmacokinetic Properties

**FIGURE 3:**
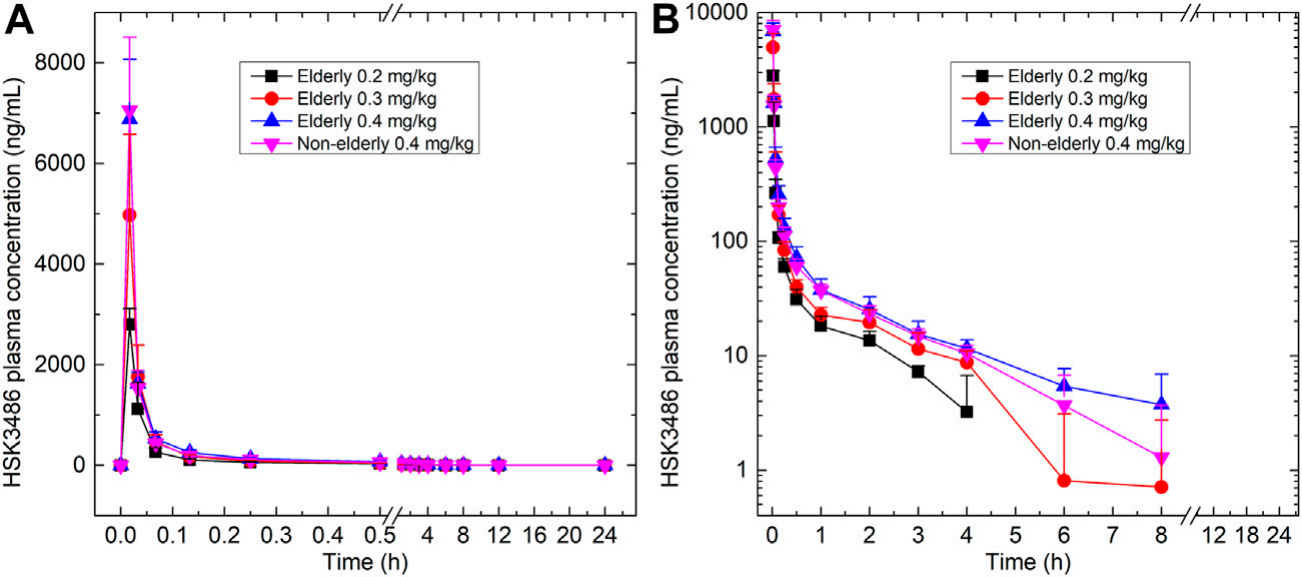
Plasma concentration-time curves for HSK3486 in **(A)** linear scale; and **(B)** semi-log scale. Data are reported as mean + standard deviation.

Mean plasma concentration-time profiles for HSK3486 in different groups are shown in Figure 3 and the pharmacokinetic parameters are shown in Table 3. After intravenous infusion of HSK3486, the time-to-peak was rapid, with rapid elimination. The plasma exposure levels (C_max_ and AUC) of the HSK3486 in the elderly groups (0.2, 0.3, and 0.4 mg/kg) increased gradually with increment of dose, while the distribution and elimination of HSK3486 showed no significant differences. The slope (β1) of the correlation between C_max_ and dose was 138% (90% CI: 109-167%), between AUC_0-t_ and dose was 123% (90% CI: 100-145%), and between AUC_0-inf_ and dose was 118% (90% CI: 96-139%). The β1 values were all greater than 1, and the 90% CIs were all not within the range of 80–125%, indicating that the C_max_ and AUC increases of HSK3486 in the dose range of 0.2–0.4 mg/kg were slightly larger than the dose increase ratio in the elderly group.

**TABLE 3:** Pharmacokinetic characteristics of HSK3486.

Parameters	Elderly 0.2 mg/kg (*N* = 8)	Elderly 0.3 mg/kg (*N* = 8)	Elderly 0.4 mg/kg (*N* = 8)	Non-elderly 0.4 mg/kg (*N* = 8)	*P*_*1*_	*P*_*2*_	*P*_*3*_	*P*_*4*_
C_max_ (ng/ml)	2802.5 (314.5)	5006.3 (1554.2)	6880.0 (1188.2)	7056.3 (1453.4)	0.000	0.002	0.776	0.000
AUC_0-t_ (ng*h/ml)	146.9 (14.37)	238.8 (40.89)	338.1 (54.99)	304.5 (32.07)	0.000	0.002	0.092	0.000
AUC_0-inf_ (ng*h/ml)	162.1 (15.88)	259.8 (42.18)	360.3 (55.17)	322.6 (32.35)	0.000	0.003	0.064	0.000
T_max_ (h)	0.02 (0.02, 0.02)	0.02 (0.02, 0.03)	0.02 (0.02, 0.02)	0.02 (0.02, 0.02)	1.000	0.168	1.000	0.407
CL (L/h)	80.0 (11.34)	74.1 (11.08)	64.3 (8.75)	76.1 (11.52)	0.476	0.705	0.035	0.042
t_1/2_ (h)	1.58 (0.25)	1.87 (0.47)	2.47 (0.49)	1.90 (0.50)	0.150	0.875	0.016	0.003
V_d_ (L)	179.2 (19.56)	200.1 (56.64)	229.0 (56.27)	206.1 (52.31)	0.278	0.807	0.356	0.259
V_dss_ (L)	80.9 (18.55)	84.9 (28.88)	94.3 (16.80)	89.1 (19.03)	0.446	0.700	0.631	0.633
MRT (h)	1.01 (0.20)	1.14 (0.35)	1.48 (0.25)	1.19 (0.29)	0.207	0.681	0.048	0.016
wn_CL ((L/h)/kg)	1.20 (0.12)	1.13 (0.20)	1.10 (0.18)	1.21 (0.13)	0.912	0.337	0.175	0.448
wn___V_d_ (L/kg)	2.72 (0.39)	3.02 (0.69)	3.87 (0.71)	3.29 (0.73)	0.089	0.414	0.081	0.010
wn_V_dss_ (L/kg)	1.22 (0.25)	1.27 (0.35)	1.60 (0.17)	1.43 (0.32)	0.141	0.280	0.246	0.050
BRPP (%)	99.2 (0.12)	99.2 (0.19)	99.2 (0.07)	99.1 (0.08)	0.165	0.422	0.078	0.304

Data are means (SD) for all except: T_max_ is median (range).

*P*_*1*_: Non-elderly 0.4 mg/kg vs Elderly 0.2 mg/kg; *P*_*2*_: Non-elderly 0.4 mg/kg vs Elderly 0.3 mg/kg; *P*_*3*_: Non-elderly 0.4 mg/kg vs Elderly 0.4 mg/kg; *P*_*4*_: Non-elderly 0.4 mg/kg vs Elderly 0.2 mg/kg vs Elderly 0.3 mg/kg vs Elderly 0.4 mg/kg; *p* < 0.05 was considered statistically significant.

C_max_: maximum observed concentration; AUC_0-t_: area under the curve from zero to last time of quantifiable concentration; AUC_0-inf_: area under the curve from the zero to infinity time; t_1/2_: terminal elimination half-life; T_max_: time to maximum concentration; CL: total clearance; V_d_: distribution volume; V_dss_: the steady state distribution volume; MRT: mean residence time; wn_CL: total clearance adjusted by weight; wn_V_d_: distribution volume adjusted by weight; wn_V_dss_: steady state distribution volume adjusted by weight; BRPP: binding rate of plasma protein.

The pharmacokinetic characteristics were similar between the 0.4 mg/kg elderly group and the 0.4 mg/kg non-elderly group. The geometric mean ratios of the pharmacokinetic parameters C_max_, AUC_0-t_, and AUC_0-inf_ of the 0.4 mg/kg elderly group relative to the non-elderly group were 98, 110, and 111%, respectively, and the 90% CIs were 82–116%, 97–125%, and 99–125%. All the 90% CIs were within the range of 80–125%, indicating that age had no significant effect on the plasma exposure of HSK3486.

The average plasma protein binding rates of the four cohorts (elderly 0.2, 0.3, and 0.4 mg/kg, and non-elderly 0.4 mg/kg) were 99.2, 99.2, 99.2, and 99.1%, respectively, and the average free fraction in plasma (fu) was 0.8, 0.8, 0.8, and 0.9%. There were no significant differences in the mean plasma protein binding rates and free fractions among the groups.

### Clinical Effects and Pharmacodynamics

**FIGURE 4:**
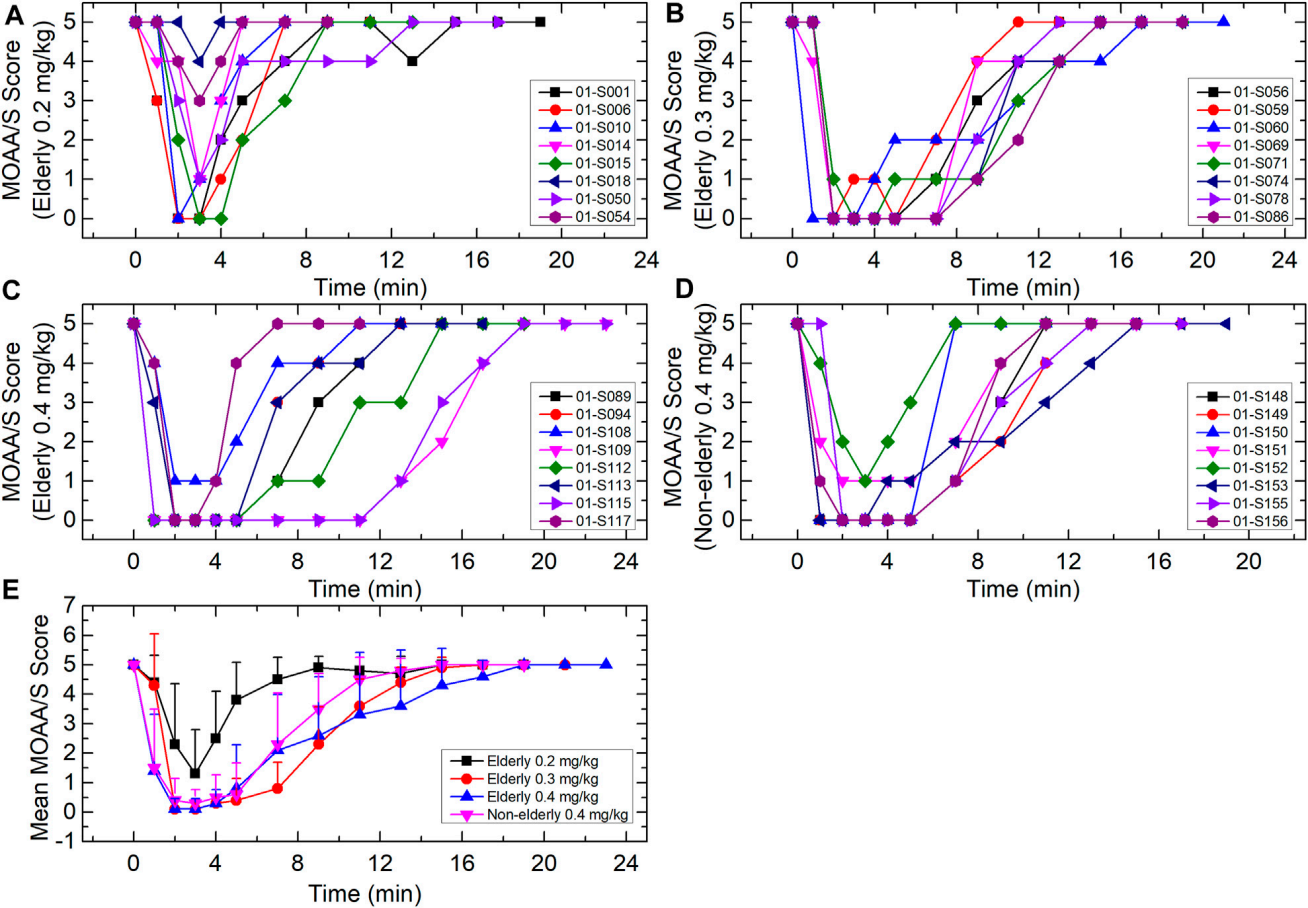
MOAA/S score-time curves for **(A)** elderly 0.2 mg/kg; **(B)** elderly 0.3 mg/kg; **(C)** elderly 0.4 mg/kg; **(D)** non-elderly 0.4 mg/kg; and **(E)** mean for each group. Data are reported as mean + standard deviation for **(E)**.

The duration of successful sedation/anesthesia induction (i.e., loss-of-consciousness time) was measured from dosing time to the first MOAA/S score ≤1 (Figure 4). The fully alert time was measured from dosing stop time to the first of three consecutive MOAA/S scores of 5. All the subjects had a MOAA/S score of five at baseline (before dosing). For the elderly 0.2, 0.3, and 0.4 mg/kg groups, the median loss-of-consciousness times were 2.54, 2.07, and 1.13 min, respectively, and the median times to full alertness were 6.02, 14.01, and 11.99 min. For the non-elderly 0.4 mg/kg group, the median loss-of-consciousness time was 1.15 min, and the median time to full alertness was 10.03 min. The loss-of-consciousness time of the elderly 0.2 mg/kg group was a little longer relative to the other three groups, while the median time to full alertness was shorter. Two subjects (S018 and S054) in the 0.2 mg/kg group were not successfully induced. The fully alert times were similar among elderly 0.3 mg/kg, elderly 0.4 mg/kg and non-elderly 0.4 mg/kg groups.

**FIGURE 5:**
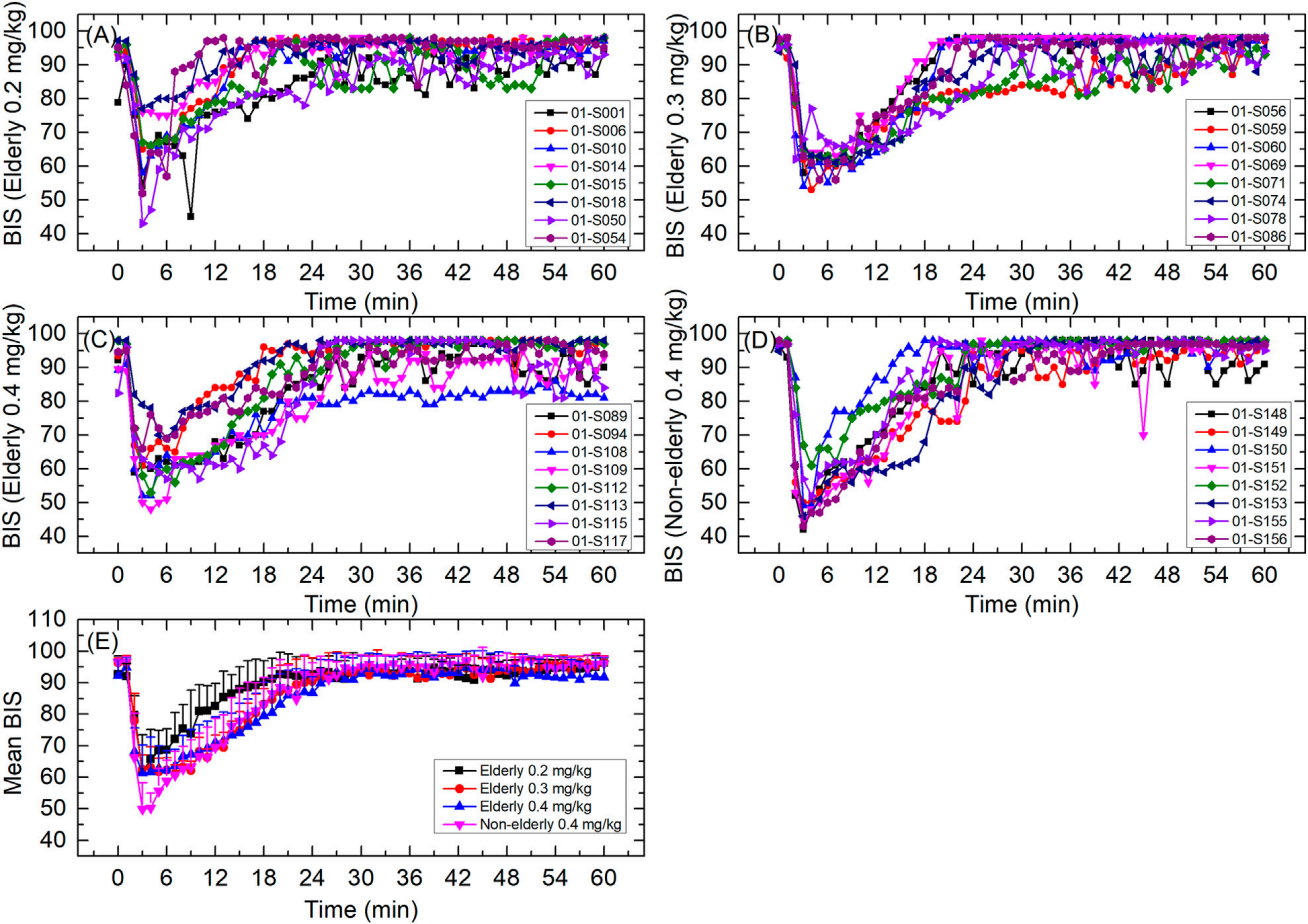
BIS curves for **(A)** elderly 0.2 mg/kg; **(B)** elderly 0.3 mg/kg; **(C)** elderly 0.4 mg/kg; **(D)** non-elderly 0.4 mg/kg; and **(E)** mean for each group. Data are reported as mean + standard deviation for **(E)**.

The individual and mean BIS curve for each group is shown in Figure 5; BIS-related parameters are shown in Table 4. For the elderly 0.2, 0.3, and 0.4 mg/kg, and non-elderly 0.4 mg/kg groups, the respective BIS parameters were as follows: mean BIS_peak_ 60.1, 58.5, 58.1, and 48.5; median T_BISpeak_ 3.00, 4.50, 3.50, and 3.00 min; mean BIS AUC_0-t_ 5,248.6, 5,067.7, 5,018.3, and 5,090.1; and mean BIS AUC_0–30 min_ 2,444.9, 2,254.1, 2,232.6, and 2,202.3. The BIS_peak_, BIS AUC_0-t_, and BIS AUC_0–30 min_ in the elderly 0.2 mg/kg group were a little higher than that of the other groups, while the BIS_peak_ in the non-elderly 0.4 mg/kg group was a little lower than the other groups. The other BIS-related parameters of all the groups were similar.

**TABLE 4:** Summary of BIS parameters.

BIS parameters		Elderly 0.2 mg/kg	Elderly 0.3 mg/kg	Elderly 0.4 mg/kg	Non-elderly 0.4 mg/kg	Total
		(*N* = 8)	(*N* = 8)	(*N* = 8)	(*N* = 8)	(*N* = 32)
BIS_peak_	Mean (SD)	60.1 (12.9)	58.5 (3.82)	58.1 (7.14)	48.5 (6.12)	56.3 (9.06)
—	Median	61.5	59.5	58.0	47.5	56.5
—	Min, Max	43, 77	53, 63	48, 69	42, 61	42, 77
T_BISpeak_, min	Mean (SD)	4.13 (2.10)	4.75 (2.05)	3.88 (1.46)	3.25 (0.46)	4.00 (1.67)
—	Median	3	4.5	3.5	3	3
—	Min, Max	3.00, 9.00	2.00, 7.00	2.00, 6.00	3.00, 4.00	2.00, 9.00
BIS AUC_0-t_	Mean (SD)	5248.6 (238.9)	5067.7 (207.6)	5018.3 (315.8)	5090.1 (190.6)	5106.2 (247.0)
—	Median	5357.8	5093.0	5020.0	5013.8	5109.5
—	Min, Max	4897.5, 5471.5	4781.5, 5354.0	4515.0, 5432.0	4877.5, 5381.5	4515.0, 5471.5
BIS AUC_0–30 min_	Mean (SD)	2444.9 (158.5)	2254.1 (107.7)	2232.6 (188.1)	2202.3 (156.8)	2283.5 (176.5)
—	Median	2494.8	2220.3	2176.8	2179.0	2234.5
—	Min, Max	2196.0, 2623.0	2,136.0, 2,422.0	2038.0, 2513.5	2028.0, 2512.5	2028.0, 2623.0

BIS_peak_: BIS peak value (the lowest BIS value); T_BISpeak_: time to BIS peak; BIS AUC_0-t_: area under the BIS curve from zero to last collection time; BIS AUC_0–30 min_: area under the BIS curve from zero to 30 min.

### Pharmacokinetic-Pharmacodynamic Association

**FIGURE 6:**
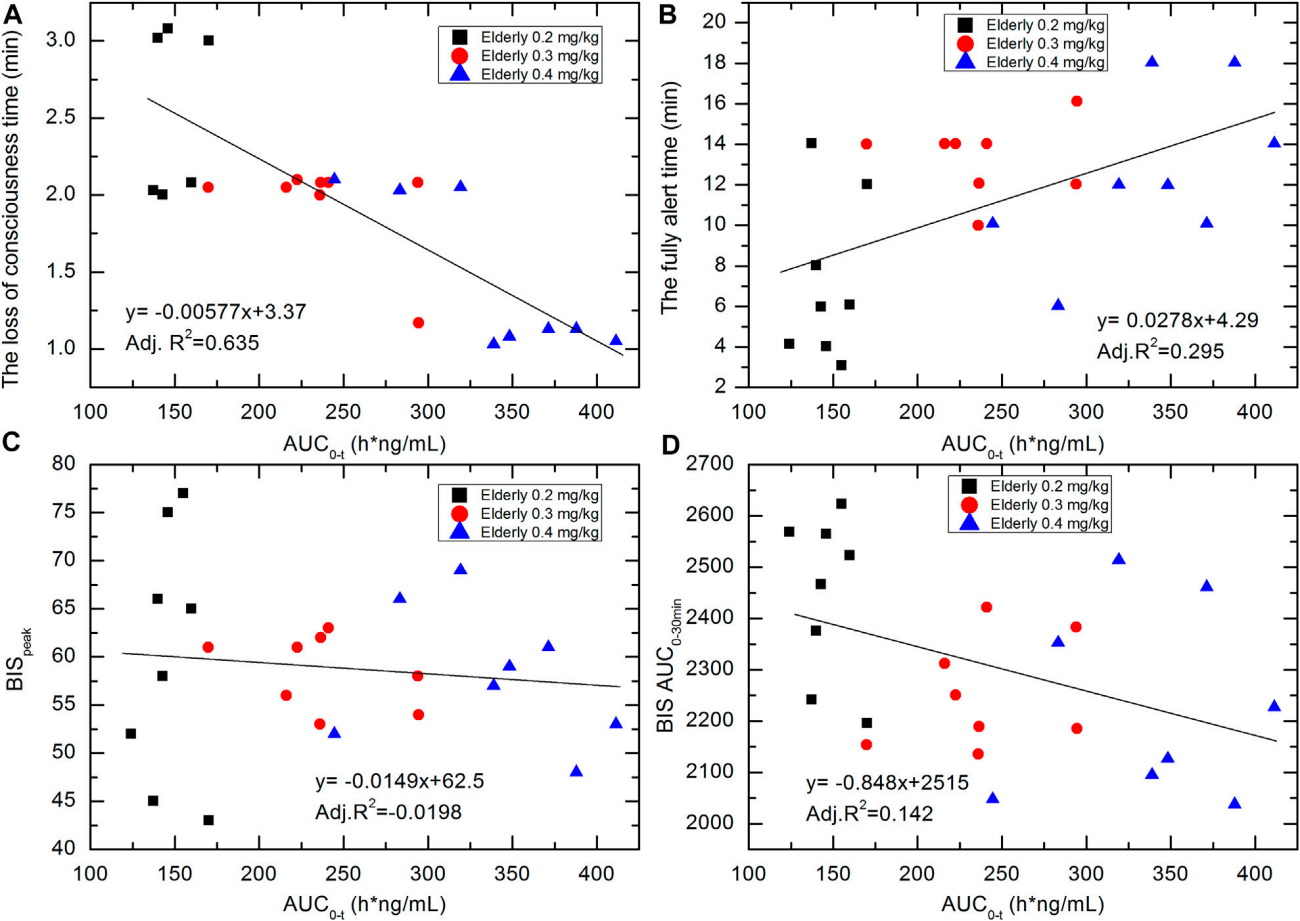
Correlation scatterplots of HSK3486 exposure level (AUC_0-t_) with **(A)** loss-of-consciousness time; **(B)** fully alert time; **(C)** BIS_peak_; and **(D)** BIS AUC_0–30 min_ for elderly 0.2, elderly 0.3, and elderly 0.4 mg/kg groups.

The pharmacokinetic-pharmacodynamic association was investigated via linear regression. The correlation scatterplots of HSK3486 exposure level (AUC_0-t_) with loss-of-consciousness time, fully alert time, BIS_peak_, and BIS AUC_0–30 min_ for the various groups are shown in Figure 6. With increases in HSK3486 exposure, the loss-of-consciousness time and BIS AUC_0–30 min_ tended to decrease, the BIS_peak_ showed a weak decreasing trend, and the time to full alertness tended to increase. The trends of C_max_ and AUC_0-inf_ were similar to that of AUC_0-t_.

## Discussion

This clinical trial investigated the effects of dose and age on the pharmacokinetic, pharmacodynamic, and safety properties of HSK3486.

In the present study, all the tested doses of HSK3486 (0.2, 0.3, and 0.4 mg/kg) in the elderly subjects, and the 0.4 mg/kg in non-elderly subjects, was well tolerated. The incidence of drug-related adverse events in the elderly group was higher than that of the non-elderly group, and was slightly higher in the elderly 0.4 mg/kg compared with the other two elderly groups. No participant discontinued the study because of a treatment-emergent adverse event related to the study drug. All adverse events were mild or moderate in intensity, and there were no death.

Propofol depresses heart rate and blood pressure through GABA_A_ receptor–mediated effects on autonomic control in the brainstem (Shirasaka et al., 2004; McDougall et al., 2008). In the present study, hemodynamics appeared to be stable throughout. There was no clinically significant change in vital sign or ECG in any group, except for 2 cases of mild transit hypotension. HSK3486 had a remarkably stable respiratory profile both in elderly and non-elderly subjects, with a low incidence of respiratory depression.

In this study, there were no spontaneous reports of pain on injection with HSK3486, which is consistent with a previous study (Bian et al., 2020). Because the most common adverse reaction associated with propofol is pain on injection, with an incidence of 25–74% in adult patients (Abad-Santos et al., 2003), the absence of pain on injection with HSK3486 is considered an improvement.

The pharmacokinetic characteristics of the 0.4 mg/kg elderly and non-elderly groups were similar. The plasma exposure of HSK3486 among the elderly groups (0.2, 0.3, and 0.4 mg/kg) increased gradually with the increase in dose, with no significant differences in distribution and elimination of HSK3486. This is consistent with a previous clinical pharmacokinetic study of HSK3486 with healthy adult subjects (Bian et al., 2020). After a single intravenous dose of 0.4 mg/kg HSK3486 in healthy adult subjects, the AUC, T_max_, t_1/2_, apparent volume of distribution during the terminal phase (Vz) and clearance were 289.2 h*ng/ml, 0.0333 h, 1.5 h, 3.0 L/kg, and 1.4 L/h/kg, respectively, which appeared comparable to corresponding parameters from our study. However, the C_max_ (at the end of 1 min intravenous bolus) was a little different, with 1,676.8 ng/ml in the literature compared with 7,056.25 ng/ml in the non-elderly subjects of the present study. Venous blood was collected in the reference study, and arterial blood was collected within 1 h post dosing in this study, the difference of C_max_ may resulted from the sampling site. The plasma concentration of HSK3486 declined in a multiphasic manner after intravenous administration. The terminal elimination half-lives of HSK3486 in this study ranged from 1.58 to 2.47 h while that of propofol was 1.5 h (Simons et al., 1988).

Consistent with the previous report (Bian et al., 2020), in the current study HSK3486 rapidly induced sedation and anesthesia, with a smooth and rapid recovery. The loss-of-consciousness time in the elderly 0.2 mg/kg group was a little longer, and the recovery time was shorter, compared with the other three groups. The time to recovery was similar among the elderly 0.3 and 0.4 mg/kg and non-elderly 0.4 mg/kg groups. The BIS_peak_ and BIS AUC of the elderly 0.2 mg/kg group were a little higher relative to the other groups.

Two subjects in the 0.2 mg/kg elderly group were not successfully induced. It has been demonstrated that the induction time of propofol depends on the dose as well as the rate of infusion (FDA, 2020). In the present study, loss-of-consciousness time/fully alert time correlated with exposure. In the elderly 0.2 mg/kg group, induction time was lower compared with the other groups, and induction failed in two subjects, perhaps due to the low dose. Yet, administration of 0.3 mg/kg to the elderly was similarly efficacious as that of the 0.4 mg/kg non-elderly control group, and with lower drug exposure. Thus, 0.3 mg/kg HSK3486 may be recommended for clinical use in elderly patients.

A limitation of the present study was that the drug effect of HSK3486 was assessed according to the MOAA/S scale, which relies on stimulation of the subject. This might bias determination of sedation and hypnosis. Secondly, for the safety reason, the present study was conducted by means of “open-label”, clinical observations were performed by clinicians and are therefore prone to subjectivity. Finally, the sample size was small. However, the preliminary results warrant clinical validation with a large sample.

In conclusion, treatment doses of HSK3486 0.2, 0.3, and 0.4 mg/kg in elderly subjects, or 0.4 mg/kg in the non-elderly, were well tolerated. The plasma exposure of HSK3486 increased gradually with increase of dose among the elderly groups. The pharmacokinetic characteristics were similar between the elderly and non-elderly, each given 0.4 mg/kg. The times to recovery of the elderly 0.3 and 0.4 mg/kg groups were comparable, which were also similar to the non-elderly control group. The loss-of-consciousness time in the elderly 0.2 mg/kg group was a little longer compared with the other groups, and the recovery time was shorter. A 0.3 mg/kg dose of HSK3486 appears suitable for clinical use in elderly patients.

## Data Availability

The original contributions presented in the study are included in the article/Supplementary Material, further inquiries can be directed to the corresponding author.
